# Challenges and Outcomes of COVID-19 Positive Neurosurgical Patients: An Institutional Experience With Emphasis on Modifications of Neurosurgical Practice

**DOI:** 10.7759/cureus.20287

**Published:** 2021-12-09

**Authors:** Binoy K Singh, Biswajit Dey, Deb K Boruah, Aishik Mukherjee, Sumit Kumar, Manoranjan Sharma, Pranjal Phukan

**Affiliations:** 1 Neurosurgery, North Eastern Indira Gandhi Regional Institute of Health and Medical Sciences, Shillong, IND; 2 Pathology, North Eastern Indira Gandhi Regional Institute of Health and Medical Sciences, Shillong, IND; 3 Radiology, Lakhimpur Medical College, Lakhimpur, IND; 4 Radiation Oncology, North Eastern Indira Gandhi Regional Institute of Health and Medical Sciences, Shillong, IND; 5 Radiology, North Eastern Indira Gandhi Regional Institute of Health and Medical Sciences, Shillong, IND

**Keywords:** postoperative outcomes, neurosurgical challenges, practice modifications, neurosurgical procedures, covid 19

## Abstract

Introduction: There has been a drastic reduction in the number of neurosurgeries performed during the COVID-19 pandemic due to a multitude of challenges prompting restructuring of neurosurgical services. The present study describes the challenges and outcomes of non-elective neurosurgical procedures done on COVID-19 positive patients along with the modifications in neurosurgical practice during the pandemic.

Methods: A retrospective study was done in the Department of Neurosurgery over a period of one year and three months. Demographic and clinical details including outcomes of the COVID-19 positive patients, who had undergone non-elective neurosurgical interventions, were collected.

Results: Ten patients (3.8%) were COVID-19 positive out of 262 neurosurgical interventions done. The age of the patients ranged from 5 days to 78 years with five males and five females. Out of the 10 patients, five were neurotrauma cases including one patient of head injury with craniovertebral junction injury. The patient with craniovertebral junction injury underwent foramen magnum decompression with C1 lateral mass-C2 pedicle screw on the right and C0-C2 pedicle screw and rod fixation on the left. The rest of the neurotrauma cases underwent craniotomy or burr-hole craniostomy followed by evacuation. Only one patient (10%) had postoperative 30-day mortality. The rest nine patients (90%) survived the post-operative 30-day mortality. The various modifications incorporated in the neurosurgical practice included categorizing the emergency room into various zones, a separate operating theatre for COVID-19 patients, limiting the number of operating members as well as minor modifications in the operating procedures.

Conclusions: The postoperative surgical outcome is favorable in COVID-19 positive patients with modifications of the existing neurosurgical practices.

## Introduction

The COVID-19 pandemic has severely impacted healthcare systems globally. Like other specialties, neurosurgery has also been affected by this pandemic. There has been a drastic reduction in the number of neurosurgical patients consulted in the outpatient department (OPD) as well as the number of surgeries performed [1]. In the Indian context, a fall of 76.25% was noticed in OPD patients and that of 70.59% in surgeries performed [1]. The surgeries performed on COVID-19 patients have become more challenging due to a multitude of factors ranging from workforce and staffing issues, procedural prioritization, intraoperative viral transmission risk, changes in postoperative care, and outcomes [2]. These challenges get compounded in resource-limited settings. While there are several studies on the restructuring of neurosurgical services and the impact of COVID-19 on workload, there is a paucity of studies on the impact of COVID-19 on the outcomes on the non-elective neurosurgical patients of COVID-19 patients [3].

The present study describes the COVID-19 positive patients undergoing non-elective neurosurgical procedures in a resource-limited setting in northeastern India. The intraoperative details, clinical course, and postoperative outcomes of the patients were analyzed.

## Materials and methods

A retrospective study was done in the Department of Neurosurgery over a period of one year and three months from July 2020 to September 2021. All COVID-19 positive pediatric and adult patients (both traumatic and non-traumatic) undergoing non-elective neurosurgical interventions were included in the study. All trauma patients admitted and managed by the departments of general surgery and orthopedics were excluded from the study.

During this period a total of 262 neurosurgical interventions were done. Out of 262 patients, 10 patients (3.8%), who were COVID-19 positive confirmed on reverse transcriptase-polymerase chain reaction (RT-PCR) or cartridge-based nucleic acid amplification test (CBNAAT) and had undergone non-elective neurosurgical interventions, were included in the study.

All demographic and clinical data were obtained by reviewing our hospital medical records. The demographic details of these COVID-19 positive cases included age and gender. Operative data included the name of the intervention and operative time. Clinical data included clinical presentation, co-morbidities, Glasgow coma scale (GCS) at presentation, radiological findings, deranged laboratory findings, length of hospital day from the day of surgery, pulmonary complications, COVID-19 status, and Glasgow outcome score or Modified Rankin score at discharge.

Continuous variables were expressed as a median. Categorical variables were summarized as counts and percentages.

## Results

A total of 262 cases had undergone neurosurgical interventions during the study period of one year and three months. Out of these cases, 10 (3.8%) cases were COVID-19 positive. The age of the patients ranged from 5 days to 78 years with a median age of 52.5 years. There were five males and five females with an M/F ratio of 1:1.

The clinical features including GCS at presentation, radiological findings, and deranged laboratory parameters are summarized in Table 1.

**Table 1:** Clinical features at presentation and radiological findings of the patients APTT: activated partial thromboplastin time; CSF: cerebrospinal fluid; ESR: erythrocyte sedimentation rate; KFT: kidney function tests; LFT: liver function tests; PT: prothrombin time; SDH: subdural hematoma.

Case	Age/ sex	Clinical presentation	Co-morbidities	GCS score	Radiological findings	Deranged laboratory findings	Diagnosis
1	78 years/male	History of trauma, unconsciousness with right hemiparesis	Hypertension, chronic kidney disease, and hepatitis	E2M5V2	Left frontotemporoparietal acute SDH with midline shift	Deranged KFT and LFT; increased D-dimer and prolonged PT	Left frontotemporoparietal traumatic acute SDH
2	50 years/male	Left hemiparesis	Hypertension and diabetes mellitus	E3M6V4	Right frontotemporal acute intracerebral hematoma with gross midline shift	Increased blood glucose; increased D-dimer, prolonged PT	Right frontotemporal acute spontaneous intracerebral hematoma
3	61 years/male	History of trivial trauma, severe headache, and right hemiparesis	Hypertension	E4M6V5	Left frontoparietal subacute SDH with midline shift	_	Subacute SDH
4	5 days/female	Swelling in the lower back with a cerebrospinal fluid leak	Premature birth	E4M6V5	Meningomyelocele lumbar region with posterior herniation of CSF filled sac containing tethered cord and nerve fibers	_	Ruptured meningomyelocele with CSF leak
5	58 years/male	History of trivial trauma and left hemiplegia	Malignant hypertension	E3M6V4	Right frontotemporoparietal subacute SDH with gross midline shift	Low hemoglobin; increased ESR	Right frontotemporoparietal subacute SDH with gross midline shift
6	13 years/ Male	History of trauma, quadriparesis, and respiratory distress	Cranio-vertebral junction anomaly	E4M6V4	(1) Atlantoaxial assimilation with hypoplastic C1; (2) traumatic atlantoaxial dislocation and atlantodentate interval 4.5 with the tip of dens compressing the cervicomedullary junction.	_	Traumatic brain injury (right thin temporal acute SDH with atlantoaxial dislocation)
7	65 years/ Female	History of trivial trauma and unconsciousness	Hypertension	E2M5V2	Bilateral frontoparietal SDH with mass effect	Low hemoglobin	Bilateral fronto-parietal SDH
8	41 years/ Female	Headache and vomiting	Malnutrition	E4M6V4	Enhancing basal exudate with ventriculomegaly	Low hemoglobin; increased ESR	Tubercular meningitis with hydrocephalus
9	50 years/ Female	Altered sensorium and left hemiplegia	Chronic glomerulonephritis	E2M5V2	Right basal ganglia bleed with cortical extension and mass effect	Deranged KFT	Right basal ganglia bleed with cortical extension
10	55 years/ Female	Sudden onset severe headache followed by unconsciousness	Hypertension	E2M5V1 (WFNS grade IV)	Right Sylvian and interpeduncular cistern bleed with cerebral edema-Fisher grade IV	Increased D-dimer and fibrinogen; prolonged PT and APTT	Right paraclinoid ruptured aneurysm

**Figure 1:**
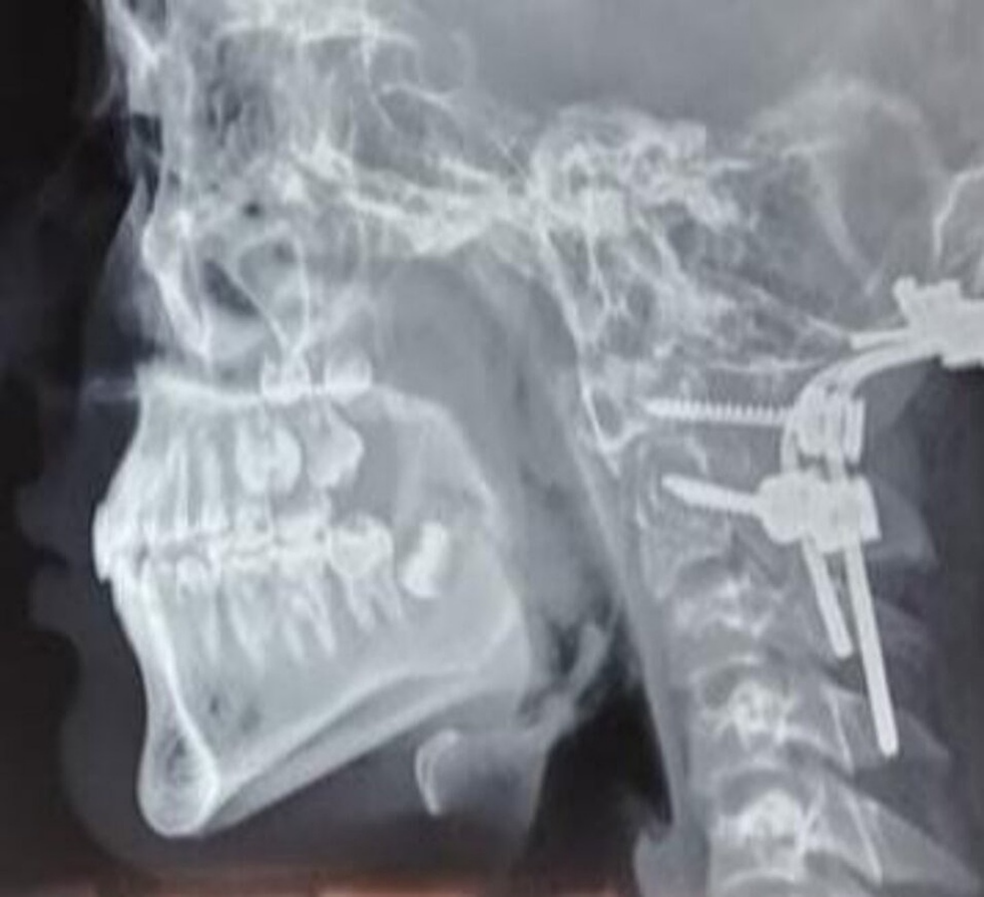
Foramen magnum decompression with C1 lateral mass-C2 pedicle screw on the right and C0-C2 pedicle screw

Out of the 10 patients, five (50%) were neurotrauma cases including one patient of head injury with craniovertebral junction injury with respiratory distress. The computed tomography (CT) chest of the patient with craniovertebral injury did not show any signs of COVID pneumonia and the patient underwent foramen magnum decompression with C1 lateral mass-C2 pedicle screw on the right and C0-C2 pedicle screw and rod fixation on the left (Figure 1). The rest of the neurotrauma cases underwent craniotomy or burr-hole craniostomy followed by evacuation.

**Figure 2:**
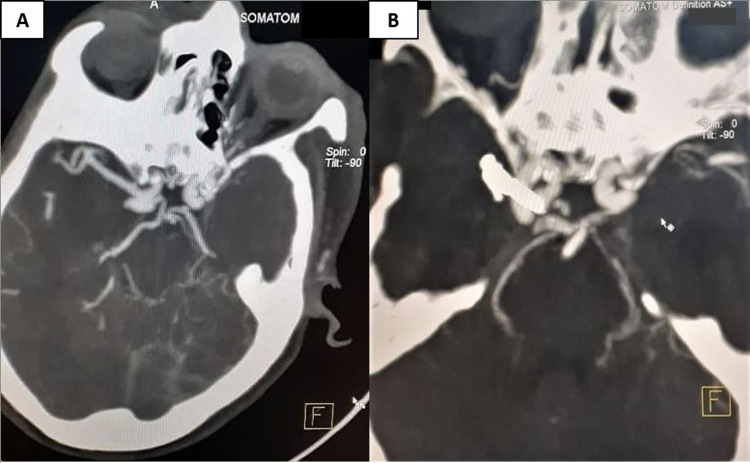
(A) Preoperative computed tomography angiography showing right paraclinoid ruptured aneurysm and (B) postoperative computed tomography angiography of the same patient showing clips in situ with complete occlusion of aneurysm

The ruptured meningomyelocele case was managed by detethering of neural structures, excision of the sac with the repair of the defect. There was one case of tubercular meningitis with hydrocephalus, which was treated by ventriculoperitoneal shunt. The patient with right basal ganglia bleed with cortical extension was managed by right frontotemporoparietal decompressive craniectomy with evacuation. There was one patient with right paraclinoid aneurysm, who had presented with right Sylvian and interpeduncular cistern bleed, was managed by extended right pterionel craniectomy with cliniodectomy with clipping of aneurysm (Figures 2A and 2B). The operative details and outcomes of the patients are summarized in Tables 1 and 2.

**Table 2:** Operative details and outcomes of the patients

Case	Neurosurgical intervention	Operative time (minutes)	Pulmonary complications	30-days mortality	Glasgow outcome score/modified Rankin score	Length of hospital stay
1	Left frontal craniotomy with evacuation	60	COVID pneumonia	Survived	1	Expired on 43^rd^ day
2	Left frontotemporoparietal craniotomy with evacuation of ICH	150	COVID pneumonia	Survived	6	Expired on 40^th^ day
3	Left frontal and parietal burr hole craniostomy with the evacuation of hematoma	90	Nil	Survived	5	40 days
4	Excision of the sac with the repair of neural placode and defect	60	Nil	Survived	5	35 days
5	Right frontal and parietal burr hole craniostomy with evacuation	90	Nil	Survived	5	40 days
6	Foramen magnum decompression with C1 lateral mass-C2 pedicle screw on right and C0-C2 pedicle screw and rod fixation on left	210	Nil	Survived	5	38 days
7	Bilateral frontoparietal burr-hole craniostomy with evacuation	150	Nil	Survived	2	21 days
8	Ventriculoperitoneal shunt	60	Nil	Survived	1	20 days
9	Right fronto-temporoparietal decompressive craniectomy with the evacuation of hematoma	180	Nil	Survived	4	32 days
10	Extended right pterionel craniectomy with anterior clinoidectomy and clipping of the aneurysm	240	Aspiration pneumonia	Expired on 25^th^ day	6	Expired on 25^th^ day

**Figure 3:**
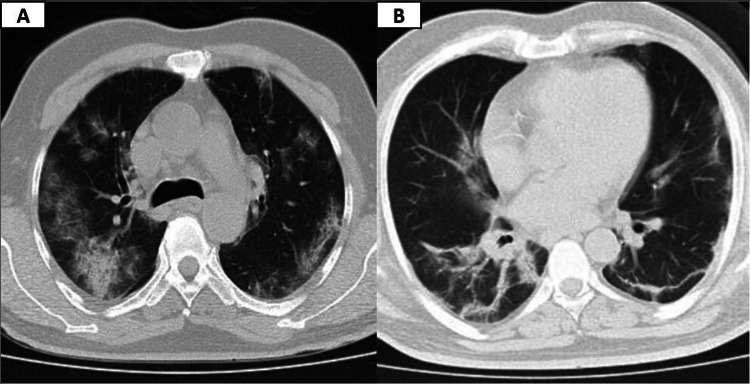
(A) High resolution computed tomography chest showing ground-glass opacity with inter and intralobular septal thickenings and (B) high resolution computed tomography showing subpleural bands and vascular dilatation

The operative duration ranged from 60 minutes to 240 minutes with a median time of 120 minutes. In the postoperative period, two patients (20%) continued to have pulmonary symptoms with persistent CT findings of COVID pneumonia (Figures 3A and 3B) and one patient (10%) had aspiration pneumonia.

In the present cohort, only one patient (10%) had postoperative 30-day mortality. The rest nine patients (90%) survived the postoperative 30-day mortality. However, two patients among them succumbed on 43rd and 40th days (Table 2).

Various modifications were introduced to tide over the challenges faced during neurosurgical interventions of the COVID-19 positive patients. These modifications are tabulated in Table 3.

**Table 3:** Modifications in neurosurgical practices PPE: personal protective equipment

Serial number	Modifications in neurosurgical practices
1.	The emergency room was categorized into three zones level A (screening area), level B (mild and moderately symptomatic COVID patients), and level C (serious COVID patients).
2.	All our patients were operated in a separate operating theatre earmarked for COVID patients with donning and doffing areas.
3.	The operating team was limited to a surgeon as a team leader with one surgical assistant, one anesthetist, one technician, one scrub nurse, and one circulatory nurse.
4.	All COVID-19 positive patients were operated on with full PPE along with N95 masks, eye-googles, and sterile gowns over the PPEs.
5.	As aerosol generation increases during craniotomy and burr-hole craniostomy, an extra face shield was used and was removed while using the microscope/loupe.
6.	Craniotomy was done under the cover of a sterile transparent bag to limit the spread of aerosols.
7.	Burr hole was made with Hudson brace perforator instead of drill Chucker.
8.	The bone flap was raised with a gentle power drill craniotome with bare saline irrigation to limit aerosol generation.
9.	Reduced visualization was an issue using eye-goggles, but it was improved by putting tape over the mask and allowing the googles to rest over the mask.
10.	Loupe with light was used for achieving hemostasis.

## Discussion

COVID-19 pandemic has resulted in an unprecedented global healthcare crisis and has put forth multiple challenges to clinicians in treating COVID-positive patients with non-COVID conditions, especially in neurosurgical practices [4,5].

In the present cohort, 10 COVID-19 patients underwent non-elective neurosurgical procedures which constituted 3.8% of all neurosurgical interventions done during the study period. So, the results are comparable to Toman et al., who had 2.2% of COVID-19 positive patients, and Singh et al. who had 2.63% positive patients with non-elective neurosurgical procedures [3,6]. A group from the United States reported the incidence of COVID-19 in neurosurgical patients to be 5.4% [7]. 

As per the institute guidelines, all patients had to undergo mandatory swab tests for SARS CoV-2. Those who required urgent neurosurgical procedures were taken up for emergency surgery considering these patients as potentially positive for SARS CoV-2. So, all these cases, which were confirmed positive or potentially positive for SARS CoV-2 were operated with full personal protective equipment (PPE) along with N95 masks, eye-googles, and sterile gowns over the PPEs. All the 10 cases in the present cohort were confirmed positive either by RT-PCR or CBNAAT.

Out of the 10 patients, five were neurotrauma cases including one case of head injury with craniovertebral junction anomaly. This patient had presented with quadriparesis and respiratory distress. There was an initial diagnostic dilemma in this patient whether respiratory distress was due to COVID pneumonia or due to cervicomedullary compression by the dens. But since the patient had no signs of COVID on the CT chest with neurological deficit, it was decided to go ahead with the plan of reduction and posterior fixation. The patient tolerated the procedure well and was extubated the same day with improvement in respiratory function. All the other cases had undergone craniotomy or burr-hole craniostomy with evacuation.

The emergency room was divided into three zones level A (screening area), level B (mild and moderately symptomatic COVID patients), and level C (serious COVID patients). All our patients were operated in a separate operating theatre earmarked for COVID patients with donning and doffing areas. The operating team was limited to a surgeon as a team leader with one surgical assistant, one anesthetist, one technician, one scrub nurse, and one circulatory nurse. None of the members of the team developed symptoms suggestive of COVID. All these guidelines were in accordance with the guidelines followed in other national institutes [8,9].

**Figure 4:**
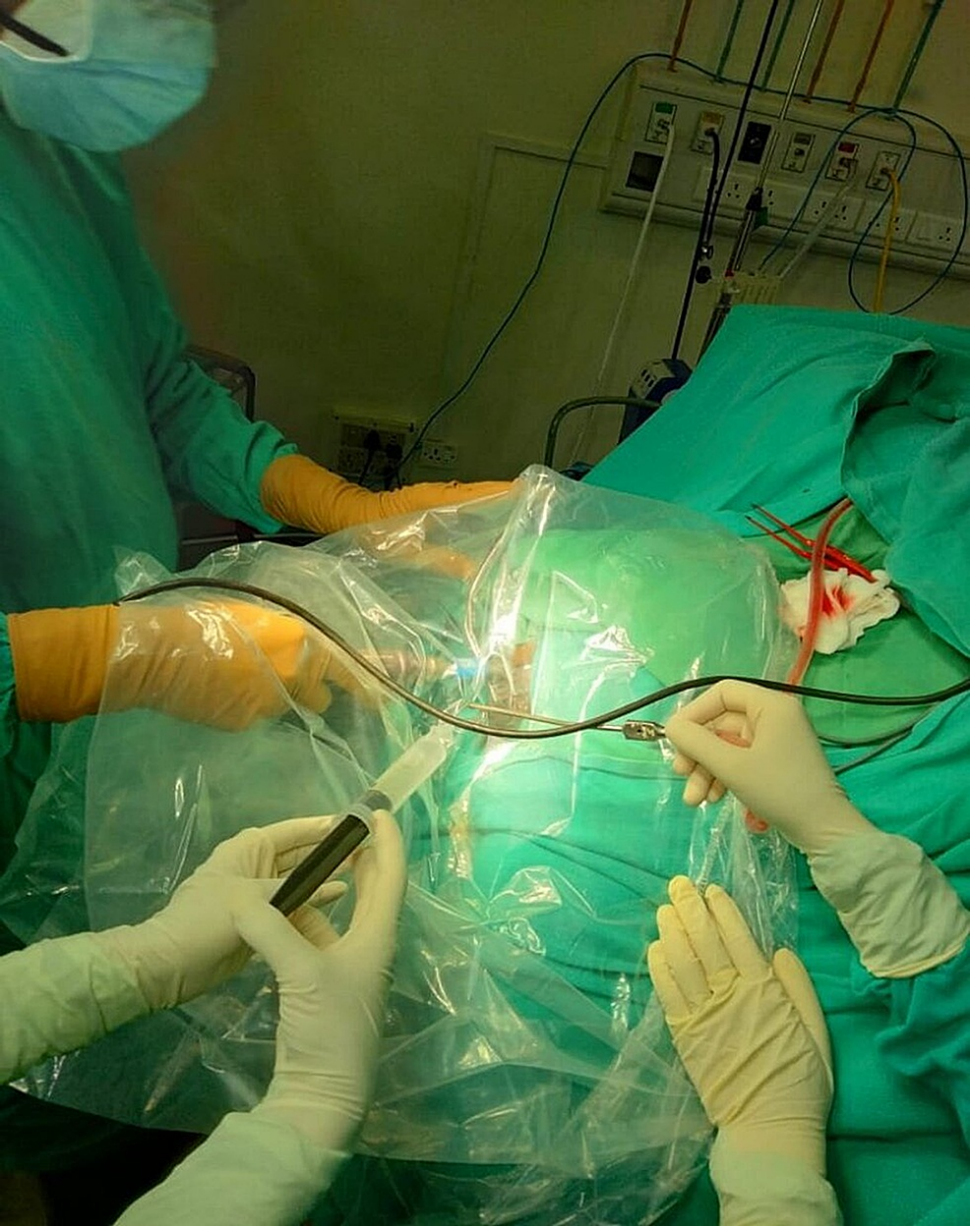
Craniotomy was done under the cover of a sterile transparent bag to limit the spread of aerosols

The operative duration ranged from 60 minutes to 240 minutes with a median time of 120 minutes. Toman et al. had a median operative time of 101.5 minutes and the authors had noticed that the average length of operation was significantly increased especially in the initial phase of the pandemic [3]. We had experienced that extra time was required due to a myriad of reasons. Craniotomy was done under the cover of a sterile transparent bag to limit the spread of aerosols (Figure 4). As aerosol generation increases during craniotomy and burr-hole craniostomy, an extra face shield was used and was removed while using the microscope/loupe. Reduced visualization was an issue using eye-goggles, but it was improved by putting tape over the mask and allowing the googles to rest over the mask. Loupe with light was used for achieving hemostasis. Burr hole was made with Hudson brace perforator instead of drill Chucker. Bone flap raised with gentle power drill craniotome with bare saline irrigation to limit aerosol generation. All the modifications in our neurosurgical practices were in concordance with other neurosurgical centers so as to maximize resource efficiency and minimize risk exposure of healthcare personnel [10,11].

During the postoperative period, two patients (20%) who had established pre-operative COVID pneumonia with multiple co-morbid conditions, continued to have pulmonary symptoms and persisted CT findings of COVID pneumonia even after immediate postoperative improvement. In spite of the aggressive management as per the COVID-19 protocols, both the patients expired after a prolonged stay in the intensive care unit (ICU). Another patient, who had a right paraclinoid ruptured aneurysm, expired due to postoperative aspiration pneumonia. The rest of the patients recovered well.

Deranged laboratory parameters are known in COVID-19 and post-COVID-19 patients [12,13]. A deranged coagulation profile, especially increased level of D-dimer and fibrinogen, as well as prolongation of prothrombin time (PT), has been associated with increased disease severity and poor outcome among COVID-19 patients [12]. In the present study, all three patients, who expired, had a deranged coagulation profile. The COVIDSurg collaborative study reported postoperative pulmonary complications in 50% of neurosurgical patients with perioperative SARS-CoV-2 infection [14]. The same group reported post-operative 30-day mortality of 18.4% [10]. All the patients in the study by Toman et al. survived the post-operative 30-day mortality and one patient succumbed on the 42nd day [3]. In the present cohort, one patient (10%) had postoperative 30-day mortality. The rest nine patients (90%) survived the post-operative 30-day mortality. However, two patients among them succumbed on 43rd and 40th days. These data assume importance in gathering evidence on how SARS-CoV-2 affects the outcome of infected neurosurgical patients although 95% of these patients have favorable outcomes [7].

## Conclusions

The postoperative surgical outcome is favorable in pediatric as well as in adult COVID-19 positive patients. Mortality increases with increased age and associated multiple co-morbidities. COVID-19 positive neurosurgical patients have to be managed with similar neurosurgical interventions as those with non-COVID-19 patients. Postoperative symptomatic neurosurgical patients with persistent pulmonary complications have high mortality in contrast to those without pulmonary complications. However, modifications of the existing neurosurgical practices may go a long way in providing effective management to COVID-19 positive patients and can prevent the spread of infections among neurosurgical health caregivers.
